# Peptides as Cosmeceuticals for Post‐Procedural Wound Healing: A Systematic Review

**DOI:** 10.1111/jocd.71283

**Published:** 2026-11-09

**Authors:** Reethi Padmanabhan, Connor Gill, Lucie Joerg, Miriam Motlak, Gerardo Guilarte, Sonal Choudhary

**Affiliations:** ^1^ University of Pittsburgh School of Medicine Pittsburgh Pennsylvania USA; ^2^ Albany Medical College Albany New York USA; ^3^ Department of Dermatology University of Pittsburgh Medical Center Pittsburgh Pennsylvania USA

## Abstract

**Background:**

Topical peptides have emerged as a promising cosmeceutical for indications ranging from hyperpigmentation to anti‐aging. However, their role in post‐procedural wound healing following aesthetic interventions remains underexplored.

**Aims:**

The aim of this study was to systematically evaluate the evidence supporting the use of topical peptide formulations in enhancing post‐procedural wound healing and patient recovery outcomes.

**Methods:**

A systematic review was conducted to identify clinical studies evaluating topical peptide application following aesthetic dermatologic procedures, including radiofrequency microneedling and non‐ablative laser therapies. Eligible studies assessed both cosmetic and therapeutic outcomes related to wound healing and recovery.

**Results:**

Six studies met inclusion criteria across three peptide systems and across ablative laser, nonablative laser, hybrid laser, and radiofrequency microneedling procedures. Peptide formulations following ablative procedures were associated with improvements in exudation, crusting, and epithelial confluence, whereas nonablative procedures demonstrated reduced erythema and improved patient‐reported recovery outcomes, including enhanced skin appearance and texture. Studies of hybrid fractional resurfacing and radiofrequency microneedling similarly reported improvements in selected early recovery outcomes.

**Conclusion:**

Topical peptide formulations show potential as adjunctive therapies for improving post‐procedural recovery following aesthetic dermatologic procedures. However, available evidence is limited by varying procedural modalities, small sample sizes, short follow‐up intervals, and restricted demographic diversity. Larger randomized controlled trials with broader representation across Fitzpatrick skin types are necessary to better define the clinical role of topical peptide cosmeceuticals in post‐procedural wound healing.

## 1 Introduction

In recent years, peptides have shown great promise as innovative bioactive agents providing cosmetic and therapeutic benefits due to their ability to selectively modulate cellular signaling pathways involved in skin homeostasis, inflammation, and extracellular matrix (ECM) remodeling. Compared with other cosmeceutical agents, peptides offer theoretical advantages including the ability to influence physiologic skin function, target specificity, and low immunogenicity [1]. These properties have inspired incorporation into topical formulations aimed at improving skin quality and recovery following dermatologic intervention.

Among the various peptide subclasses, signal peptides have received particular attention for their ability to stimulate fibroblast activity and ECM turnover. Signal peptides promote collagen and elastin synthesis, enabling potential reduction of photoaging signs and improvement of skin texture [2, 3]. Moreover, these effects have prompted investigation into the role of signal peptide formulations in supporting post‐procedural wound healing, as these procedures involve controlled skin injury and barrier disruption with subsequent inflammation.

Aesthetic procedures for anti‐aging, including ablative and nonablative lasers as well as radiofrequency microneedling, are widely used to target wrinkles, dyschromia, and textural abnormalities. However, these procedures consistently induce acute inflammatory responses characterized by edema, erythema, and crusting, resulting in patient discomfort and procedural downtime [4]. Standard post‐procedure treatment involves bland ointments and emollients to augment barrier repair, but these serve as passive approaches that do not directly modulate the underlying mechanisms of wound healing.

Topical peptide–based cosmeceuticals have therefore been proposed as adjunctive therapies to regulate post‐procedural healing through targeted biologic mechanisms. Several peptide systems have been evaluated in this context, including ECM signal peptide complexes, regenerative multi‐component peptides (MCPs) with broader pathway activity, and angiopoietin‐derived peptides that may influence vascular and inflammatory responses. Despite increasing clinical use, evidence supporting these interventions remains limited, and no prior systematic review has specifically evaluated topical peptide–based formulations in dermatologic wound healing.

In this systematic review, we synthesize available evidence on topical peptide formulations for post‐procedural wound healing, stratified by procedure type. Specifically, we summarize findings related to the fibroblast‐stimulating TriHex Technology system, the regenerative MCP system, and the endothelial junction–stabilizing *Q*‐peptide, and evaluate their mechanisms, clinical efficacy, and patient‐reported recovery outcomes following ablative laser, non‐ablative laser, and radiofrequency microneedling aesthetic dermatologic procedures.

## 2 Materials and Methods

A comprehensive literature search was conducted across PubMed, Cochrane Library, and Web of Science to identify studies evaluating topical peptide formulations for post‐procedural wound healing following aesthetic dermatologic procedures. The search strategy incorporated terms related to peptides, topical or cosmeceutical applications, and wound healing or aesthetic dermatologic procedures. Complete search strategies for each database are provided in Supplementary Table 1.

Included studies were peer‐reviewed original clinical investigations (randomized controlled trials (RCTs), non‐randomized controlled studies, prospective cohorts, split‐face/within‐subject studies, and relevant clinical pilot trials) spanning from January 2015 to December 2025. These studies involved human participants undergoing dermatologic or aesthetic procedures (e.g., laser resurfacing, microneedling, dermabrasion, chemical peel, fractional or ablative laser) that resulted in controlled skin injury or barrier disruption, along with use of peptide‐containing topical formulations applied post‐procedure for wound healing, skin regeneration, or recovery. Comparator groups included standard wound care, vehicle control, or other intervention lacking peptides. Eligible studies were required to report at least one relevant clinical or histologic endpoint: wound closure, erythema resolution, time to re‐epithelialization, collagen synthesis, scar improvement, or patient satisfaction.

Studies consisting of exclusively preclinical in vivo, in vitro, or ex vivo studies were excluded, along with studies investigating non‐peptide (e.g., hyaluronic acid, growth factors, vitamins) without peptide content. Preclinical findings reported within otherwise eligible clinical studies were considered as supportive mechanistic evidence but were not independently included in clinical synthesis. Studies on chronic wounds, ulcers, or surgical incisions not related to dermatologic or aesthetic procedures were excluded. Reviews, meta‐analyses, conference abstracts without full text, non‐peer‐reviewed material, patents, or promotional reports were also excluded.

Study selection and data extraction were performed independently by two reviewers, with discrepancies resolved through discussion and consensus. Extracted variables included study design, sample size, participant characteristics, Fitzpatrick skin type when reported, procedural modality, peptide formulation, comparator, treatment duration, follow up period, clinical outcomes, patient‐reported outcomes, adverse events, and principal findings. The study selection process was conducted in accordance with the Preferred Reporting Items for Systematic Reviews and Meta Analyses (PRISMA) framework. A PRISMA flow diagram summarizes the study selection process (Figure 1).

**FIGURE 1:**
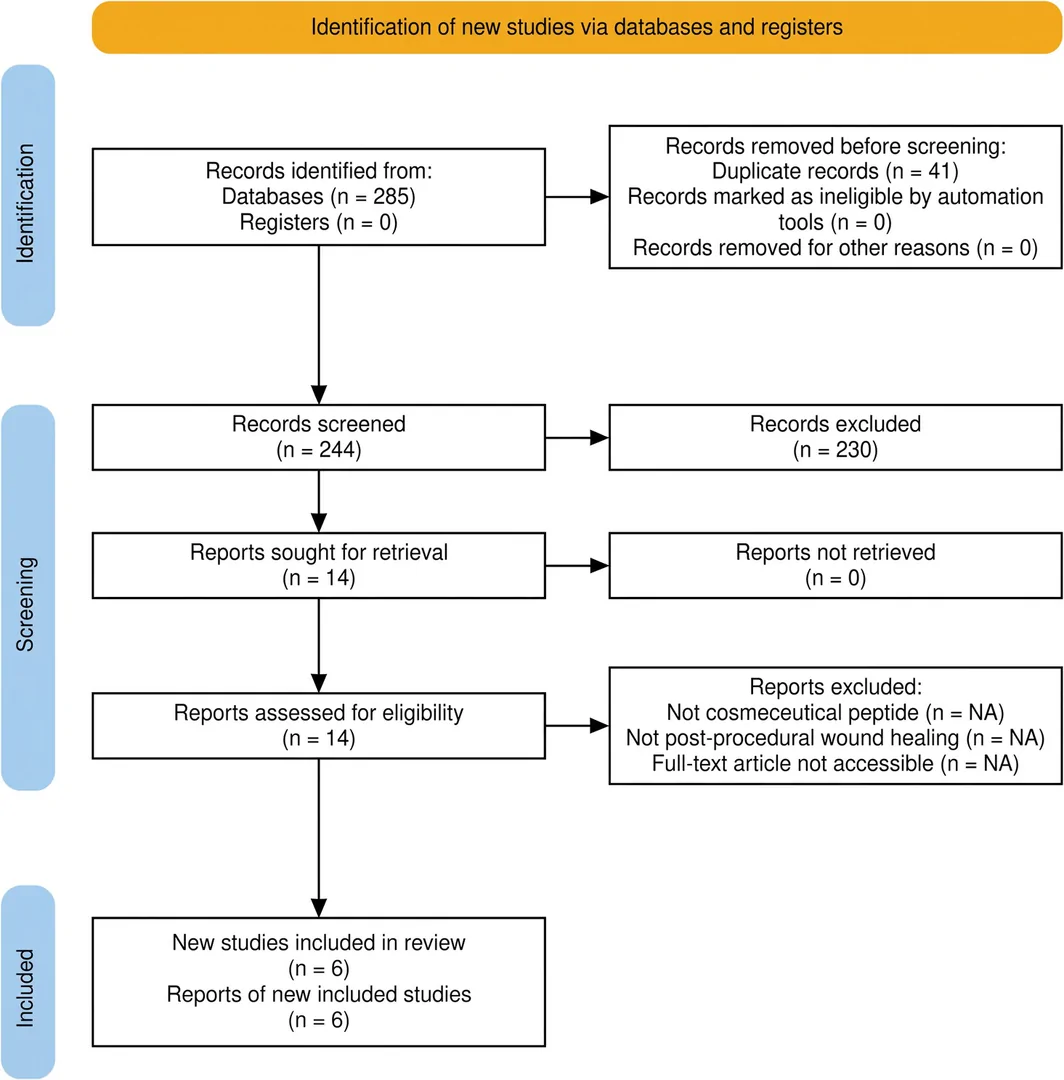
PRISMA flow diagram of study selection for included articles. Diagram depicting the identification, screening, and inclusion of studies evaluating topical peptides in post‐procedural wound healing.

Given differences in extent and mechanism of cutaneous injury across aesthetic procedures, included studies were stratified by procedural modality for qualitative synthesis. Studies were categorized as ablative laser, nonablative laser, hybrid laser, or radiofrequency microneedling. Outcomes were interpreted within these procedural categories. Given a small number of included studies and heterogeneity in procedural modality, peptide formulations, comparators, and reported outcomes, a quantitative meta‐analysis was not performed.

## 3 Results

The database search yielded 285 records, of which 41 duplicates were removed. After thorough abstract screening of 244 articles, 14 full‐text articles were assessed for eligibility. Six articles met all inclusion and exclusion criteria and were included in the final synthesis (Table 1).

**TABLE 1:** Summary of included studies evaluating topical peptides in post‐procedural wound healing.

Study (Author, year)	Peptide type	Study design	Procedural category	Procedure	Sample size (n)	Intervention vs Control	Key outcomes	Main findings
Vanaman Wilson [5]	TriHex	RCT, single‐blind	Ablative laser	Fractionated CO2	15	TriHex vs. standard care	Erythema, healing scores, symptoms	↓ Erythema ↓ Exudation Improved healing at 1 week
Liao [6]	MCP	RCT	Ablative laser	Erbium/CO_2_ wound model	20	MCP versus AHO versus none	Wound healing, epithelialization	↑ Epithelial confluence ↓ Crusting
Wang [7]	TriHex	Split‐face RCT	Nonablative laser	1927 nm fractional thulium	20	TriHex versus moisturizer	Skin quality, erythema, edema	↓Erythema/edema Improved tone, texture
Qian [8]	MCP	Split‐face RCT	Nonablative laser	Fractional laser	34	MCP versus AHO	Anti‐aging outcomes, TEWL	Improved wrinkles, tone Similar TEWL
Nelson [9]	TriHex	Prospective	Hybrid laser	Fractional laser	5	TriHex versus standard care	Redness, roughness	↓ Erythema ↓ Roughness/crusting
Giorgio [10]	Q‐peptide	Split‐face RCT	RFMN	RF Microneedling	8	Q‐peptide versus petrolatum	Erythema, edema, satisfaction	↓Erythema/edema ↑ Patient satisfaction

Abbreviations: AHO, Aquaphor Healing Ointment; TEWL, transepidermal water loss.

Two studies evaluated ablative laser injury, two evaluated nonablative fractional laser resurfacing, one evaluated hybrid fractional laser surfacing, and one evaluated radiofrequency microneedling.

### 3.1 Ablative Laser Procedures

Two studies evaluated topical peptide formulations following ablative laser injury [5, 6]. Vanaman Wilson et al. evaluated TriHex technology following fractionated CO_2_ laser resurfacing, while Liao et al. evaluated 10% MCP after erbium/CO_2_ ablative laser.

TriHex Technology is a peptide‐based topical platform designed to support extracellular matrix (ECM) remodeling and enhance cutaneous regenerative capacity. This system combines palmitoyl tripeptide‐1 and palmitoyl hexapeptide‐12 peptides to stimulate tropoelastin and procollagen production processes for post‐procedural structural repair [5] Vanaman Wilson et al. reported that patients treated with TriHex displayed significantly reduced erythema and exudation 3 days after CO_2_ resurfacing, as well as superior blinded‐investigator healing scores at 1 week (*p* = 0.01), reflecting a measurable improvement in inflammatory resolution and wound healing [5]. Patients also reported decreased tenderness, burning, and stinging throughout the first two postoperative weeks, suggesting reduced inflammation‐associated discomfort following ablative facial resurfacing.

The second ablative laser therapy study investigated the effects of the multi‐component reaction peptide (MCP), acetyltrifluoromethylphenyl valylglycine, which had been shown to support postprocedural wound healing by enhancing skin barrier repair kinetics following tape stripping and attenuating inflammation and post‐inflammatory hyperpigmentation in prior preclinical in vitro and ex vivo studies [6].

In a randomized controlled trial, Liao et al. investigated post‐laser wound healing efficacy in 20 healthy men and women with Fitzpatrick skin Types I–III, who received either a topical 10% MCP formulation, Aquaphor Healing Ointment (AHO), or no treatment following erbium/CO_2_ ablative laser–induced mini‐zone wounds on the volar forearm [6]. Based on investigator assessment, 10% MCP was associated with improved wound healing parameters compared with controls, including significantly reduced crusting and scabbing at Day 7 and increased epithelial confluence at Days 11–14 (*p* < 0.05). No significant differences in treatment‐related erythema or edema were observed between groups, and the formulation was well tolerated with no reported adverse events. Unlike studies with less disruptive procedures, this ablative wound model allowed direct assessment of epithelial recovery and wound‐specific endpoints like crusting and epithelial confluence.

### 3.2 Nonablative Fractional Laser Procedures

Two studies evaluated peptide formulations after nonablative fractional laser resurfacing [7, 8]. In contrast to wound‐specific ablative injury endpoints, these studies evaluated inflammatory signs, skin quality, and barrier function.

Wang et al. also evaluated TriHex serum, this time following non‐ablative fractional laser, and observed reduced erythema and edema at 1 week and superior investigator‐rated global skin quality scores [7]. Furthermore, lentigines and skin tone reached statistical significance (*p* = 0.0086 and *p* = 0.0030, respectively) [7]. These findings suggest that the primary observed benefits of TriHex involved inflammatory recovery and skin quality outcomes over wound closure.

Another study investigating wound healing following non‐ablative laser therapy was conducted by Qian et al., who led a split‐face randomized controlled trial evaluating the healing and anti‐aging effects of MCP versus AHO in 34 White female participants following full face non‐ablative fractional laser resurfacing [8]. MCP‐supported post‐procedural wound healing to a degree comparable with AHO; however, MCP was associated with significantly greater improvements in multiple anti‐aging outcomes, including crow's feet, infraorbital wrinkles, surface roughness, skin tone evenness, radiance, and dyschromia. MCP and AHO performed similarly in measures of epidermal thickness and skin barrier restoration, assessed by transepidermal water loss (TEWL) (*p* > 0.05). Participants reported stinging, burning, pain, pruritus, tightness, tingling, erythema, and dryness without significant differences between groups. Thus, unlike the epithelial healing findings observed in the ablative MCP model, the nonablative study primarily demonstrated improvements in aesthetic recovery without evidence of superior barrier restoration.

### 3.3 Hybrid Fractional Laser Resurfacing

One study evaluated post‐procedural wound healing following hybrid fractional resurfacing, a process combining both ablative and non‐ablative components [9]. Authors Nelson et al. evaluated TriHex peptides following the hybrid fractional laser and noted reductions in redness, roughness, and crusting during the first postoperative week, with four of five participants preferring the peptide‐treated side. Despite a small sample size, these findings suggest a potential improvement in early recovery following a procedure producing mixed cutaneous injury.

### 3.4 Radiofrequency Microneedling

One study evaluated peptide therapy following radiofrequency microneedling (RFMN), a minimally invasive procedure that delivers targeted radiofrequency energy to the dermis to stimulate new ECM protein production and produces a pattern of mechanical and thermal injury distinct from laser resurfacing. In a randomized, double‐blind split‐face study, Giorgio et al. examined the effect of an Angiopoietin‐1‐derived peptide, the QHREDGS (Q‐peptide), on eight female participants. This study reported significant improvements in wound appearance and feel from both clinician and subject perspectives, along with reduced erythema, edema, and purpura from image analysis measures [10]. Compared to the side with petrolatum ointment only (Cerave healing ointment), clinicians preferred the *Q*‐peptide side at both 2 days (*p* = 0.001) and 7 days post‐procedure (*p* = 0.026), while subjects similarly reported better look and feel at 2 days (*p* = 0.015) and 7 days (*p* = 0.026). Image analysis also reported faster recovery to baseline skin texture (*p* = 0.034), as well as reduced scabbing and microneedling insertion marks at Day 2 (*p* = 0.023).

## 4 Discussion

This systematic review identified six clinical studies evaluating three topical peptide systems following aesthetic procedures that produced varying degrees of cutaneous injury through different mechanisms. Peptide‐based formulations were generally associated with improvements in post‐procedural recovery, although the benefits varied depending on procedural modality. Given that ablative resurfacing, nonablative resurfacing, hybrid fractional laser, and radiofrequency microneedling induce different patterns of epidermal and dermal injury, clinical wound healing endpoints were evaluated within the context of each procedural modality.

Following ablative injury, discussed endpoints were most specific to wound healing, whereas nonablative fractional studies highlighted improvements in aesthetic recovery and overall skin quality. In the fractionated CO_2_ resurfacing study, TriHex treatment was associated with reduced exudation [5], while MCP treatment after erbium/CO_2_ ablative laser noted reduced crusting and greater epithelial confluence [6]. These endpoints are directly relevant to recovery following epidermal disruption. Meanwhile, in nonablative laser treatment studies, MCP demonstrated greater improvement in several aesthetic parameters like infraorbital wrinkles and skin evenness, but no improvement over AHO in barrier restoration [8]. TriHex application following non‐ablative laser therapy also showed reduction in general inflammatory appearance, including reduction of edema and erythema [7]. These findings suggest that the benefit of peptide formulations may depend on the underlying injury: wound and epithelial recovery may be relevant following ablative treatment, whereas inflammatory resolution and recovery quality may be more informative following nonablative procedures.

Hybrid fractional resurfacing and radiofrequency microneedling warrant separate consideration as neither produces an injury pattern equivalent to ablative or nonablative laser therapies alone. TriHex was associated with improvements in early redness and roughness following hybrid fractional treatment, whereas angiopoietin‐1‐derived *Q*‐peptide improved clinician and patient assessed recovery and image analysis measures after RFMN.

Despite these encouraging findings, several limitations constrain the interpretation and generalizability of this evidence. Although these findings may support potential utility across multiple procedural settings, observed benefits differ according to the degree and mechanism of injury, as do expected post‐procedural sequelae. For instance, recovery assessments following ablative laser resurfacing emphasized exudation, crusting, tenderness, burning, and stinging, all findings reflecting a greater degree of epidermal disruption. In contrast, nonablative fractional laser studies predominantly assessed transient inflammatory and sensory findings including erythema, edema, itching, and discomfort related to treatment. Hybrid resurfacing involved similar early redness and roughness, whereas radiofrequency microneedling was associated with a distinct recovery profile characterized by erythema, edema, purpura, scabbing, and microneedling insertion marks. These findings should be distinguished from true treatment‐related adverse outcomes; notably, no serious or significant treatment‐related adverse events were reported in the included studies that explicitly reported safety outcomes.

Also, mechanistically, these peptide systems are heterogeneous. TriHex contains signal peptides proposed to support extracellular matrix remodeling, whereas MCP has demonstrated broader effects on barrier repair, fibroblast activity, and inflammatory pathways in preclinical models. Angiopoietin‐1‐derived *Q*‐peptide has been explored for its potential role in endothelial stabilization and regenerative signaling. These mechanisms may contribute to different recovery aspects within each procedural modality, although the current clinical evidence is insufficient to establish a relationship between each mechanism and observed clinical outcomes.

Additional limitations include small study populations and limited demographic diversity. All included trials enrolled small cohorts, with some studies enrolling fewer than ten participants and predominantly White, female subjects. Follow‐up periods were abbreviated, typically ranging from 7 to 30 days, limiting assessment of long‐term epithelial remodeling, collagen synthesis, and pigmentation. These constraints are especially relevant for patients with Fitzpatrick IV‐VI skin tones, who may be more vulnerable to post‐inflammatory hyperpigmentation and scarring. Additionally, some studies were financially supported by dermatology companies, introducing potential sponsorship bias.

Taken together, current literature supports a potential adjunctive role for topical peptide based formulations in improving early post‐procedural recovery and patient experience following aesthetic dermatologic procedures. However, additional independently funded trials with large, diverse patient populations and extensive follow‐up intervals are necessary to better define clinical utility, durability, and applicability of these agents in post‐procedural wound healing.

## 5 Conclusion

Across the available clinical literature, topical peptide formulations, including TriHex Technology, multi‐component reaction peptides, and angiopoietin‐1‐derived peptide have been examined as adjunctive options to support post‐procedural wound healing following aesthetic dermatological procedures. While evidence suggests these formulations may support post‐procedural recovery, the observed benefits differ according to the degree and mechanism of procedural injury.

Following ablative laser treatment, topical peptide application was associated with wound‐specific outcomes including reduced exudation and crusting and increased epithelial confluence. Nonablative fractional procedures instead predominantly demonstrated improvements in erythema, edema, skin quality, and perceived patient recovery. Findings following hybrid fractional resurfacing and RFMN similarly suggest potential benefits in early recovery but have limited additional supporting data.

These findings overall support interpreting peptide efficacy within the context of procedural injury. Current evidence remains constrained by small sample sizes, short follow‐up windows focused primarily on acute healing, limited demographic diversity, and potential sponsorship bias in some trials.

Future investigations should prioritize larger randomized controlled trials with longer follow‐up and broader representation in participant skin tone across Fitzpatrick skin types to better evaluate which peptide formulations may produce meaningful clinical benefits for specific patterns of cutaneous injury.

## Author Contributions

Reethi Padmanabhan: conceptualization, methodology, data curation, writing. Connor Gill: data curation, writing, editing. Lucie Joerg: data curation, writing, editing. Miriam Motlak: mentorship, methodology, editing. Gerardo Guilarte: mentorship, methodology, editing. Sonal Choudhary: supervision, methodology, critical revision of manuscript.

## Funding

The authors have nothing to report.

## Ethics Statement

This study is a systematic review of previously published literature and did not involve direct human subject participation or identifiable patient data. Institutional review board approval was not required.

## Conflicts of Interest

The authors declare no conflicts of interest.

## Supporting information


**Supplementary Table 1** Database search strategies used for the systematic review.

## Data Availability

Data sharing not applicable to this article as no datasets were generated or analysed during the current study.
