# Supplementary material for: Peptides as Cosmeceuticals for Post‐Procedural Wound Healing: A Systematic Review
Source: J Cosmet Dermatol. 2026 Nov 9;25(11):e71283. doi: 10.1111/jocd.71283 (PMC13747723; doi:10.1111/jocd.71283)
Supplement: Supplementary file 1 [file JOCD-25-e71283-s001.docx]

**Supplementary Table 1. Database search strategies used for the systematic review**

| **Database** | **Complete Search Strategy** | **Records Identified** |
| --- | --- | --- |
| PubMed | (("Peptides"[MeSH] OR peptide* OR oligopeptide* OR polypeptide* OR "copper peptide*" OR "GHK-Cu" OR matrikine*) AND ("Cosmeceuticals"[MeSH] OR cosmeceutical* OR "skin care" OR "topical agent*" OR "cosmetic dermatology") AND ("Wound Healing"[MeSH] OR "wound healing" OR "skin regeneration" OR "laser resurfacing" OR microneedling OR "chemical peel" OR dermabrasion)) | 197 |
| Cochrane | (peptide* OR oligopeptide* OR polypeptide* OR bioactive NEXT peptide* OR copper NEXT peptide* OR GHK OR GHK NEXT Cu OR matrikine* OR synthetic NEXT peptide* OR biomimetic NEXT peptide*) AND (cosmeceutical* OR cosmetic* OR skin NEXT care OR topical NEXT agent* OR anti NEXT aging OR skin NEXT rejuvenation OR skin NEXT repair OR regenerative NEXT dermatology OR skin NEXT product*) AND (wound NEXT healing OR skin NEXT regeneration OR collagen NEXT synthesis OR scar NEXT formation OR epithelialization OR laser NEXT resurfacing OR fractional NEXT laser OR microneedling OR chemical NEXT peel OR dermabrasion OR burn OR injury OR cutaneous NEXT wound OR post NEXT procedure OR post NEXT laser OR postoperative NEXT healing) | 38 |
| Web of Science | ((peptide* OR oligopeptide* OR polypeptide* OR "copper peptide*" OR "GHK-Cu" OR matrikine*) AND (cosmeceutical* OR "skin care" OR "topical peptide*" OR "cosmetic dermatology") AND ("wound healing" OR "skin regeneration" OR "laser resurfacing" OR microneedling OR dermabrasion OR "chemical peel")) | 50 |

*Searches were restricted to studies published from January 2015 to December 2025. Abbreviation: MeSH, Medical Subject Headings*
